# Biochemical-Anthropometric Indices Assessing Liver Steatosis Identified in Elastography in Children with Metabolic DysFunction-Associated Steatotic Liver Disease

**DOI:** 10.3390/nu18182996

**Published:** 2026-09-13

**Authors:** Aleksandra M. Motkowska, Anna Lebensztejn, Bartosz Tuchlinski, Kinga Trochimczyk, Marta Flisiak-Jackiewicz, Anna Bobrus-Chociej, Magdalena Rogalska, Beata Cudowska, Katarzyna Zdanowicz, Dariusz M. Lebensztejn

**Affiliations:** 1 Students’ Scientific Association Affiliated with the Department of Pediatrics, Gastroenterology, Hepatology, Nutrition, Allergology and Pulmonology, Medical University of Bialystok, Waszyngtona Street 17, 15-274 Bialystok, Poland; 2 Department of Pediatrics, Gastroenterology, Hepatology, Nutrition, Allergology and Pulmonology, Medical University of Bialystok, Waszyngtona Street 17, 15-274 Bialystok, Poland; 3 Department of Infectious Diseases and Hepatology, Medical University of Bialystok, Zurawia Street 14, 15-540 Bialystok, Poland

**Keywords:** fatty liver index, hepatic steatosis index, lipid accumulation product, metabolic dysfunction-associated steatotic liver disease, liver steatosis, controlled attenuation parameter, obesity, children

## Abstract

**Background**: Metabolic dysfunction-associated steatotic liver disease (MASLD) is the most common cause of liver pathology in children. Hepatic fat content can be assessed by a transient elastography, but this method is not widely used. Therefore, the aim of this study was to evaluate the usefulness of a simple, surrogate biochemical-anthropometric indices of liver steatosis: FLI (Fatty Liver Index), HSI (Hepatic Steatosis Index), and LAP (Lipid Accumulation Product) in children with MASLD. **Methods**: The retrospective study included a group of 103 obese/overweight children. MASLD was diagnosed according to criteria developed by a multisociety statement published in Journal of Pediatric Gastroenterology and Nutrition in 2024. Liver steatosis (controlled attenuation parameter/CAP/) was assessed in elastography. HSI, FLI, and LAP were calculated according to their mathematical formulas. ROC analysis and multivariate logistic regression were performed to calculate the power of the studied indices in diagnostics of liver steatosis in children with MASLD. **Results**: MASLD was diagnosed in 72 children (70%). Children with MASLD had significantly higher levels of ALT, AST, GGT, uric acid, value of BMI, waist circumference, HOMA-IR, and indices HSI, FLI, LAP compared to the group of non-hepatopathic controls (n = 31). HSI, FLI, and LAP significantly correlated with CAP. All indices identified MASLD in the ROC analysis (AUC = 0.77, 0.76, 0.68 resp.). However, only HSI remained a significant independent predictor of hepatic steatosis in overweight/obese children. **Conclusions**: Although HSI, FLI, and LAP can be considered as useful biochemical-anthropometric indicators of MASLD in obese/overweight children, only HSI remained an independent predictor of hepatic steatosis in this group of children.

## 1. Introduction

The prevalence of overweight and obese children in the pediatric population is steadily increasing and becoming a significant global health problem. According to the WHO, in 2024, 35 million children under the age of five were overweight. While just 2% (31 million) of young people (aged 5–19) were obese in 1990, by 2022, 8% (160 million) of children and adolescents were living with obesity [1]. Recently Zhang et al. published a systematic review and meta-analysis including data from 154 countries; they reported that 22.2% children were overweight and overall prevalence of obesity was 8.5% [2]. Obesity, especially visceral obesity, is a component of metabolic syndrome, which also includes hypertension, dyslipidemia, and abnormal glucose tolerance [3,4]. In particular, young-onset overweight and obesity is strongly associated with short- and long-term complications involving multiple organs, including insulin resistance and type 2 diabetes, atherosclerosis, left ventricular hypertrophy, early cardiovascular events, kidney disease, and metabolic-dysfunction-associated steatotic liver disease (MASLD) [5]. Currently, MASLD is the most common liver disease in children and adults: 7–14% of children and adolescents and 38% of adults have MASLD [6]. Currently, MASLD is diagnosed according to the criteria developed by numerous scientific societies, which include confirmation of liver steatosis and one of five pediatric cardiometabolic criteria [7,8]. A key element in the diagnosis of MASLD is confirmation of the presence of hepatic steatosis. Liver biopsy with morphological assessment is still considered the reference standard, but due to its invasiveness (risk of complications), sampling bias, and interobserver variability, it is now less commonly used in children to assess ectopic fat accumulation in the liver [9]. 

On the other hand, non-invasive ultrasonography can detect only moderate/severe steatosis (>33%); it further suffers from significant interobserver variability [10]. Computer tomography, due to its use of ionizing radiation, is not recommended for the diagnosis of hepatic steatosis in the pediatric population [11]. Magnetic resonance methods (high proton density fat fraction, proton spectroscopy, elastography) are sensitive methods (detecting 5% fat fraction), but are expensive and still not widely available [12]. Transient elastography (Controlled Attenuation Parameter—CAP) is increasingly used in both adults and children to assess hepatic fat content, but cut-offs for both steatosis diagnosis and grading are still being validated [13]. 

There is urgent need to investigate noninvasive, low-cost, simple biomarkers or algorithms. Increasing evidence suggests that such algorithms may be useful in evaluating hepatic steatosis. They include standard liver-related blood tests (AST, ALT, GGT), blood tests associated with hyperlipidemia (triglycerides) and anthropometric parameters (body mass index, waist circumference). Therefore, the aim of the study was to evaluate the usefulness of simple, surrogate biochemical-anthropometric indices: fatty liver index (FLI) [14], hepatic steatosis index (HSI) [15], and lipid accumulation product (LAP) [16], in identifying liver steatosis assessed in transient elastography in overweight/obese children with MASLD.

## 2. Materials and Methods

The study protocol was approved by the bioethics committee of Medical University of Bialystok, Poland (No: APK.002.373.2023). All aspects of this study were made in compliance with the Declaration of Helsinki.

The retrospective study included a group of 103 obese or overweight children (BMI ≥ 85%), aged 7–18 years (median 12 years) newly admitted to the Department due to suspected liver disease (elevated serum ALT activity and/or hepatomegaly and/or “bright liver” in ultrasound). Children with selected metabolic liver diseases (e.g., Wilson’s disease, alpha-1-anitrypsin deficiency), autoimmune hepatitis, celiac disease, cystic fibrosis, HCV, and HBV infections (potential MASLD overlap) were excluded from the analysis. History of hepatotoxic drug use was also an exclusion criterion. MASLD was diagnosed in 72 children (70%) based on the multisociety statement published in Journal of Pediatric Gastroenterology and Nutrition in 2024 [8]. The criteria for diagnosis were based on the presence of hepatic steatosis on imaging studies, along with the fulfillment of at least one of the five cardiometabolic criteria [7]. The control group consisted of 31 (30%) non- hepatopathic children with excessive body weight who did not meet the diagnostic criteria for MASLD.

### 2.1. Anthropometric Measurements

Body Mass Index (BMI) was calculated by dividing body weight by square meter of height (kg/m^2^). BMI ≥ 85th percentile according to centile charts was considered as excessive body weight. Waist circumference (WC) was measured at the narrowest point of the abdomen between the last rib and the iliac crest. Children with WC ≥ 95th percentile according to appropriate age- and sex-appropriate centile charts were declared overweight. Hip circumference (HC) was taken by measuring at the widest point of the buttocks. Body Waist Hip Ratio (WHR) was calculated dividing waist circumference by hip circumference [17].

### 2.2. Biochemical Parameters

All sera were collected from patients after an overnight fast. The biochemical parameters: alanine aminotransferase (ALT), aspartate aminotransferase (AST), gamma-glutamyl transferase (GGT), bilirubin, cholesterol, glucose, insulin, uric acid (UA), triglycerides (TG) high-density lipoprotein cholesterol (HDL-C), and low-density lipoprotein cholesterol (LDL-C) were determined using standard laboratory methods. The Homeostatic Model Assessment of Insulin Resistance (HOMA-IR) was calculated according to a mathematical formula: HOMA-IR = (fasting insulin [µIU/mL] × fasting glucose [mg/dL]/22.5) [18].

### 2.3. Assessment of Hepatic Steatosis

Assessment of hepatic steatosis was performed by one certified hepatologist using transient elastography (FibroScan, Echosens, Paris, France) after a minimum of four hours of fasting prior to the testing. The controlled attenuation parameter (CAP), showing the severity of liver steatosis, was measured for each child. CAP > 249 dB/m was considered the cut-off point for the diagnosis of hepatic steatosis [13].

### 2.4. Steatosis Indices

Hepatic Steatosis Index (HSI), Fatty Liver Index (FLI), and Lipid Accumulation Product (LAP) were calculated according to the mathematical formulas:

FLI = (e0.953 × loge(TG) + 0.139 × BMI + 0.718 × loge(GGT) + 0.053 × WC − 15.745)/(1 + e0.953 × loge(TG) + 0.139 × BMI + 0.718 × loge(GGT) + 0.053 × WC − 15.745) × 100 [14],

HSI = 8 × (ALT/AST ratio) + BMI (+2, if female; +2, if diabetes mellitus) [15],

LAP = (WC − 58) × TG for girls; LAP = (WC − 65) × TG for boys [16].

### 2.5. Statistical Analysis

Statistical analysis was performed using Statistica software (the Polish version of Statistica 13.0, TIBCO Software Inc., Palo Alto, CA, USA), for Windows PCs. Data were expressed as median and interquartile range (Q1–Q3). Comparisons between children with and without MASLD were performed using the Mann–Whitney U test. Correlations between continuous variables were assessed using Spearman’s rank correlation coefficient. Receiver operating characteristic (ROC) curve analysis was performed to evaluate the diagnostic performance of FLI, HSI, and LAP to identify MASLD. The optimal cut-off values were determined using the Youden index. Univariate logistic regression analysis was performed to identify factors associated with MASLD. Variables showing significant associations and without evidence of substantial multicollinearity were subsequently included in the multivariable logistic regression model. A two-sided *p* value < 0.05 was considered statistically significant.

## 3. Results

MASLD was diagnosed in 69.9% (n = 72) of the studied group. The age of the children in the MASLD group was not significantly different from the age of the children in the non-hepatopathic group. Comparison of selected anthropometric, biochemical, and biochemical-anthropometric indices of a liver steatosis between MASLD group (n = 72) and non-hepatopathic controls (n = 31) highlighted important differences (Table 1). Children with MASLD had significantly higher levels of ALT, AST, GGT, insulin, uric acid, and values of HOMA-IR, waist circumference, hips circumference, and BMI. Value of calculated indices: FLI, his, and LAP were statistically higher in children with MASLD than in non-hepatopathic controls. There was no statistically significant difference in LAP values between the genders (*p* = 0.383).

Spearman correlation analysis was carried out in order to assess the relationship between the calculated biochemical-anthropometric indices and controlled attenuation parameter (CAP) on elastography. Significant positive correlation with CAP was obtained for all studied indices: HSI (r = 0.425, *p* < 0.001), FLI (r = 0.42, *p* < 0.001), and LAP (r = 0.33, *p* = 0.008).

**Figure 1:**
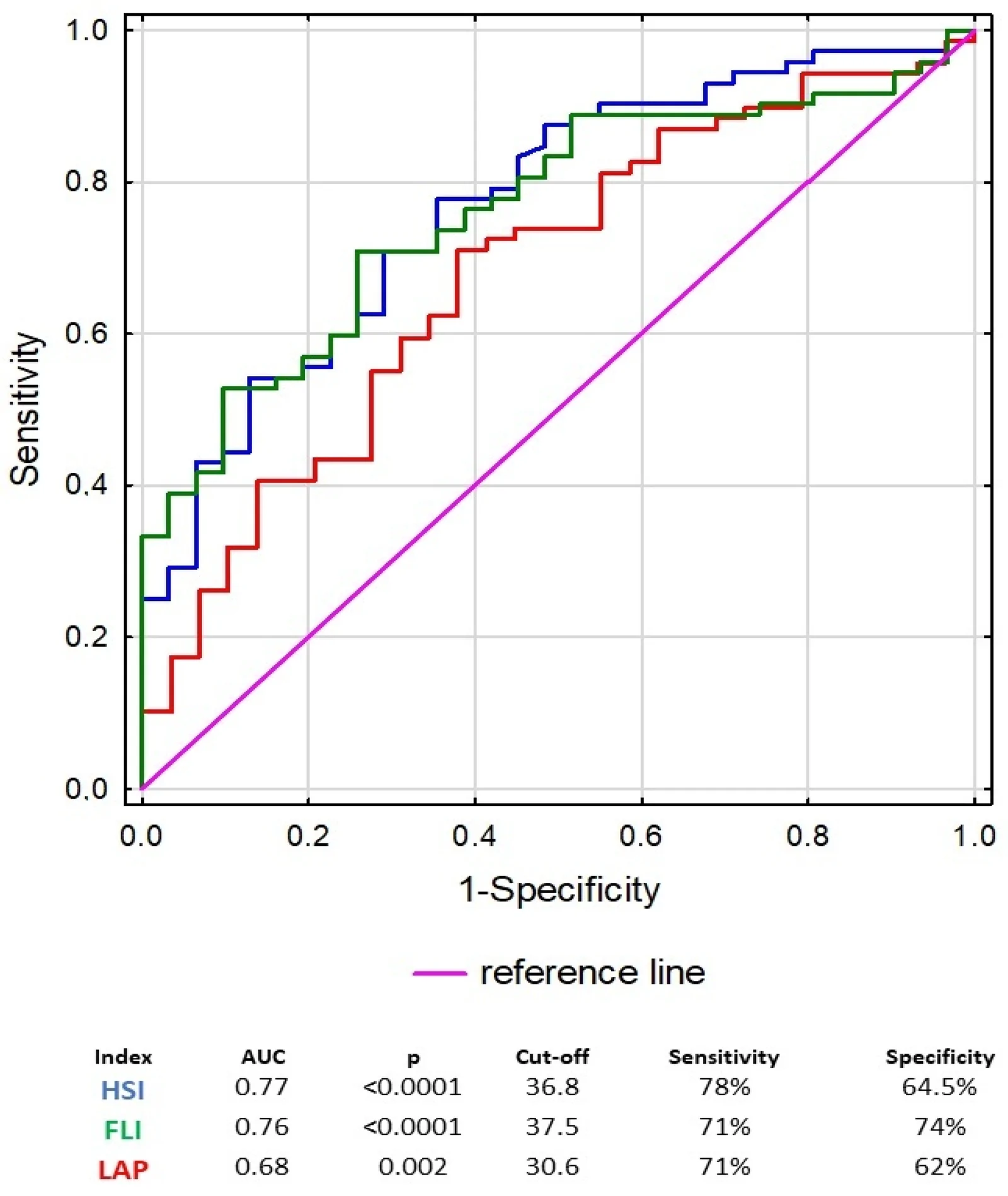
Receiver operating characteristics (ROC) curve of the ability of examined indices to differentiate patients with liver steatosis from patients without steatosis. Area under curve (AUC), cut-off, sensitivity, and specificity of the assessed biochemical-anthropometric indices in identifying liver steatosis. HSI—hepatic steatosis index, LAP—lipid accumulation product, FLI—fatty liver index.

ROC analyses were performed to calculate the power of the studied indices in identifying hepatic steatosis (Figure 1). The ability of HSI, LAP, and FLI to differentiate children with liver steatosis from those without MASLD was significant. The highest predictive values for identifying liver steatosis had HSI and FLI (AUC = 0.77, AUC = 0.76, resp.) (Figure 1).

In the univariate logistic regression analysis, several metabolic indices showed significant associations with hepatic steatosis. Higher HSI, LAP, and FLI were strongly related to the presence of steatosis. In addition, insulin, HOMA-IR, and UA were also significant predictors of hepatic steatosis. Because FLI showed strong correlations with both LAP (r = 0.80) and HSI (r = 0.73), it was excluded from the multivariate model to reduce multicollinearity. The multivariate logistic regression therefore included HSI, LAP, HOMA-IR, and UA (Table 2). In this model, only HSI remained a statistically significant independent predictor of hepatic steatosis in overweight/obese children (OR = 1.152; 95% CI: 1.026–1.292; *p* = 0.016).

## 4. Discussion

MASLD is currently the most common chronic liver disease in children and adolescents and represents the hepatic manifestation of metabolic dysfunction. Accurate identification of hepatic steatosis is therefore essential for establishing the diagnosis and selecting children who require further evaluation. Furthermore, the severity of steatosis, regardless of the degree of liver fibrosis, is associated with systemic inflammation and has prognostic value as a predictor of progressive liver disease (in severe cases, liver-related mortality) as well as cardiovascular disease, metabolic syndrome, and diabetes [19]. Although imaging techniques such as transient elastography with CAP provide a non-invasive assessment of hepatic steatosis, pediatric-specific CAP cut-off values, and grading thresholds are still undergoing validation. Therefore, there is a need for simple, inexpensive, and non-invasive tools that could facilitate the identification of children at increased risk of MASLD.

As expected, in our study, children with MASLD differed significantly from those without hepatic steatosis in several anthropometric and metabolic parameters. They had higher BMI, waist and hip circumferences, insulin concentrations, HOMA-IR, uric acid levels, and liver enzyme activities. These findings are consistent with the current understanding of MASLD as the hepatic manifestation of metabolic dysfunction, and are in agreement with previous pediatric studies demonstrating that hepatic steatosis is closely associated with obesity, insulin resistance, and other metabolic abnormalities [20,21,22].

To address the clinical need, several non-invasive biochemical and anthropometric indices have been proposed for the identification of hepatic steatosis. Such tools could be particularly useful in primary care and outpatient settings, where access to transient elastography remains limited. In adults, several non-invasive indices, including the HSI, FLI, and LAP, have demonstrated acceptable diagnostic performance in detecting hepatic steatosis. Nevertheless, current American Association for the Study of Liver Diseases (AASLD) guidelines do not recommend the routine use of blood-based non-invasive algorithms for the assessment of hepatic steatosis because the available evidence remains insufficient to support their widespread clinical application [19]. Consequently, further validation studies, particularly in pediatric populations, are still warranted. Therefore, considering the limited evidence regarding the applicability of these indices in children and adolescents, we evaluated the performance of HSI, FLI, and LAP in identifying hepatic steatosis, using transient elastography with CAP as the imaging-based reference, in an overweight and obese pediatric population.

In our study, HSI values were significantly higher in children with MASLD compared with those without hepatic steatosis. Moreover, HSI demonstrated a moderate positive correlation with CAP values obtained by transient elastography, suggesting that this index reflects the amount of hepatic fat accumulation. Among the evaluated non-invasive indices, HSI showed the highest diagnostic performance, with an AUROC of 0.77. However, the observed discriminative ability was moderate and does not support the use of HSI as a standalone diagnostic tool for MASLD. The optimal cut-off value of 38.8 showed moderate ability to discriminate between children with and without hepatic steatosis, with 78% sensitivity and 65% specificity. Furthermore, in univariate logistic regression analysis, higher HSI values were significantly associated with the presence of MASLD. Importantly, after adjustment for other metabolic parameters, including LAP, HOMA-IR, and uric acid, HSI remained the only independent predictor of MASLD in our pediatric cohort.

HSI was first introduced by Lee et al. in 2010 [15] as a simple non-invasive tool for identifying adult patients with hepatic steatosis. The index was developed using routinely available clinical parameters, including ALT/AST ratio, BMI, sex, and the presence of diabetes mellitus, combined into a mathematical formula. In the original study, two cut-off values were proposed: an HSI value below 30 suggested a low probability of NAFLD, whereas a value above 36 supported the diagnosis of hepatic steatosis. Subsequent studies aimed to validate the diagnostic performance of HSI in various populations. However, when interpreting these results, it is important to consider the heterogeneity between studies, including differences in disease definitions (NAFLD, MAFLD, and more recently MASLD) as well as the methods used for the assessment of hepatic steatosis. Most studies evaluating HSI have been conducted in adult populations. In one of the most recently published studies, which included patients with ultrasound-confirmed MASLD and liver biopsy as the reference standard, histological steatosis grade was positively associated with HSI values. Moreover, although the primary aim of the study was to evaluate the diagnostic performance of ultrasound-derived fat fraction (UDFF), HSI demonstrated diagnostic accuracy comparable to UDFF for identifying moderate and severe hepatic steatosis [23]. Similar findings were reported by Li et al. in a cohort of adults with MAFLD diagnosed using vibration-controlled transient elastography. In multivariable logistic regression analysis, HSI was associated with the presence of MAFLD, together with abdominal volume index, waist-to-height ratio and body roundness index [24].

To the best of our knowledge, data on the diagnostic performance of HSI in pediatric MASLD remain scarce. In a recently published study including children with MASLD diagnosed using transient elastography with CAP, HSI demonstrated excellent diagnostic accuracy, with an AUC of 0.91. An HSI cut-off value of 30 provided high sensitivity (96%) but only moderate specificity (63%), whereas increasing the cut-off to 40 substantially improved specificity (95%), making this threshold more suitable for confirming MASLD. Notably, among children and adolescents with normal alanine aminotransferase levels, HSI maintained good diagnostic performance [25]. In our cohort, HSI also demonstrated acceptable diagnostic performance; however, the AUC was lower (0.77). The optimal cut-off value identified in our study (38.8) was close to the higher threshold proposed in the previous pediatric study. However, given the moderate discriminative performance observed in our cohort, this threshold should be considered population-specific and should not be regarded as a universally applicable diagnostic cut-off. Nevertheless, unlike the previous study, we evaluated a single optimal cut-off value derived from ROC analysis. Further evidence supporting the usefulness of HSI in pediatric populations was provided by a study including children, in which hepatic steatosis was assessed using CAP. Similar to our findings, HSI showed a moderate positive correlation with CAP values, indicating that increasing HSI values were associated with greater hepatic fat accumulation [13]. The potential usefulness of HSI in pediatric populations was also confirmed by another study evaluating children with suspected MAFLD. HSI values were significantly associated with the presence of MAFLD in both sexes and remained a significant predictor in multivariable analyses. Moreover, HSI demonstrated good diagnostic performance, with AUC values of 0.89 in boys and 0.84 in girls [26].

The second non-invasive index evaluated in our study was FLI. Originally developed by Bedogni et al., FLI was designed as a simple algorithm for predicting hepatic steatosis in the general adult population [14]. In our study, FLI values were also significantly higher in children with MASLD than in obese controls without hepatic steatosis. FLI demonstrated moderate discriminative performance for identifying hepatic steatosis, with an AUC of 0.76. As for HSI, this level of diagnostic performance is insufficient to support the use of FLI as a standalone diagnostic tool. In univariate logistic regression analysis, higher FLI values were significantly associated with the presence of MASLD. However, FLI was not included in the multivariable model because of its strong correlation with HSI and LAP. 

In contrast to the wide evidence available in adults, studies evaluating FLI in children with MASLD remain limited. In the study by Ferraioli et al., which evaluated several non-invasive markers against CAP, FLI showed a moderate positive correlation with CAP values, comparable to that observed for HSI. Moreover, FLI demonstrated acceptable diagnostic accuracy for detecting hepatic steatosis (AUC = 0.76), which was very similar to that observed in our cohort [13]. Similar findings were reported in a cohort of overweight and obese children from Sri Lanka. NAFLD was diagnosed by ultrasonography. FLI values were significantly higher in children with NAFLD than in those without hepatic steatosis and remained an independent predictor of NAFLD in multivariable analysis. The diagnostic performance of FLI was moderate (AUC = 0.69), with an optimal cut-off value of 30 for identifying NAFLD [27]. The most recent pediatric study, based on data from the National Health and Nutrition Examination Survey (NHANES) cohort and an independent real-world clinical cohort, further confirmed the diagnostic utility of FLI in children and adolescents. FLI demonstrated excellent diagnostic performance for identifying MASLD, with AUC values of 0.91 in the NHANES cohort and 0.93 in the external validation. The authors proposed two diagnostic thresholds for FLI: a lower cut-off of 20 to support screening and a higher cut-off of 50 to confirm MASLD. For the identification of severe MASLD, the optimal FLI cut-off increased to 60. Importantly, FLI maintained excellent diagnostic performance even among children with normal ALT concentrations [25]. In contrast, the diagnostic power of FLI in our cohort was lower. This discrepancy may be explained by differences in the study populations, inclusion criteria, and the fact that we derived a single optimal cut-off value. Additional evidence supporting the utility of FLI was provided by a study including children aged 9–11 years with a wide range of BMI values (19–96 percentile). In this cohort, FLI was identified as a significant predictor of MAFLD irrespective of sex and demonstrated good diagnostic performance, with AUC values of 0.85 in girls and 0.95 in boys. Moreover, FLI showed significant positive correlations with other metabolic parameters associated with hepatic steatosis [26]. Compared with our cohort, the study population was younger and included children across the BMI spectrum rather than exclusively overweight and obese patients, which should be considered when comparing the diagnostic performance of FLI between studies.

The last non-invasive parameter evaluated in our study was LAP. Interestingly, this index was not originally developed to assess hepatic steatosis but was proposed as a simple anthropometric-biochemical marker of excessive lipid accumulation and cardiometabolic risk [16]. Several years later, its potential application was extended to the prediction of hepatic steatosis in adults, where it demonstrated promising diagnostic performance [28]. In our study, LAP values were significantly higher in children with MASLD than in those without hepatic steatosis. LAP was also positively correlated with CAP values, although this correlation was weaker than those observed for HSI and FLI. Although LAP demonstrated a statistically significant ability to discriminate between children with and without hepatic steatosis, its discriminative performance was limited compared with the other evaluated indices, with an AUC of 0.68 suggesting that LAP may be of complementary rather than primary diagnostic value. In the univariable logistic regression analysis, higher LAP values were significantly associated with the presence of MASLD. However, after adjustment for HSI, HOMA-IR, and uric acid in the multivariable model, LAP was not an independent predictor of MASLD. Nevertheless, studies in adults suggest that LAP may serve as a simple screening tool for hepatic steatosis [24,29,30,31,32]. In one study, its diagnostic performance was the highest among individuals aged 18–50 years [24], whereas another study demonstrated that LAP was independently associated with NAFLD in normal-weight adults and showed better predictive accuracy than BMI or waist circumference alone (AUC = 0.81) [31]. Conversely, in a recent study comparing several non-invasive indices with ultrasound-derived fat fraction, LAP demonstrated lower diagnostic performance than HSI, particularly in patients with moderate and severe hepatic steatosis [23]. These findings are in line with our results. To the best of our knowledge, only one pediatric study has specifically evaluated the diagnostic performance of LAP for hepatic steatosis. Özcabı et al. demonstrated that LAP was significantly higher in children with NAFLD than in those without hepatic steatosis, and achieved moderate diagnostic accuracy (AUC = 0.70). The authors proposed a cut-off value of 42.7 for identifying NAFLD and concluded that LAP may represent a simple and practical tool for screening hepatic steatosis in children with obesity [33]. Notably, the cut-off identified in our cohort was lower (28), which may reflect differences in study populations, age, sex distribution, obesity status, diagnostic definitions, and the reference method used to assess hepatic steatosis. These findings are consistent with our results, as LAP was also significantly higher in children with MASLD and showed a significant association with hepatic steatosis. However, in our cohort, its diagnostic performance was lower than that of HSI and FLI, suggesting that LAP may be of complementary rather than primary diagnostic value. Most pediatric studies evaluating LAP have focused on its usefulness as a marker of metabolic disturbances including impaired glucose metabolism [34], hypertension [35], and increased cardiometabolic risk [36,37], rather than on its role in the diagnosis of hepatic steatosis.

From a clinical perspective, the potential utility of these surrogate indices may lie primarily in screening rather than in the definitive diagnosis of MASLD. HSI and FLI, based on routinely available anthropometric and biochemical parameters, may help identify children at increased likelihood of hepatic steatosis and support decisions regarding further imaging-based assessment. This may be particularly relevant in primary care or outpatient settings where access to transient elastography or other advanced imaging methods is limited. However, given the moderate discriminative performance observed in our cohort, these indices should be considered complementary screening tools rather than substitutes for transient elastography, magnetic resonance imaging (MRI), or other imaging-based methods used for the definitive assessment of hepatic steatosis.

This study has several strengths. First, unlike population-based studies, our cohort consisted exclusively of overweight and obese children at increased risk of MASLD, representing a clinically relevant population in whom non-invasive screening tools are most likely to be applied. Second, hepatic steatosis was assessed using transient elastography with CAP, providing a non-invasive imaging-based assessment within the study cohort. Third, we simultaneously evaluated and directly compared the diagnostic performance of three commonly used non-invasive indices (HSI, FLI, and LAP) within the same cohort. Finally, the use of multivariable logistic regression allowed us to assess the independent diagnostic value of the evaluated indices while accounting for other metabolic factors. Several limitations should also be mentioned. This was a retrospective study conducted in a relatively small, single-center cohort, which may limit the generalizability of our findings. We did not perform sex-specific analyses or account for pubertal stage. We identified a single optimal cut-off value for each index based on ROC analysis, whereas the diagnostic performance of multiple predefined thresholds was not evaluated. An additional limitation is the use of CAP as the imaging-based reference for hepatic steatosis. Although CAP is a non-invasive and widely used method for assessing liver fat, pediatric-specific cut-off values and grading thresholds are still undergoing validation. Therefore, the diagnostic performance and cut-off values identified in our study should be interpreted with caution and require confirmation in larger pediatric studies using validated reference methods.

## 5. Conclusions

The growing prevalence of childhood obesity and its metabolic complications, including MASLD, represents an increasing public health problem. In this context, there is a need for simple, inexpensive, and widely available non-invasive tools that may help identify children at increased risk of hepatic steatosis and support decisions regarding further diagnostic evaluation. In our cohort, all three evaluated indices showed some ability to distinguish children with and without hepatic steatosis, with HSI showing the best overall performance. However, their diagnostic performance was moderate, and these indices should not be used as standalone diagnostic tools or as a replacement for imaging-based methods. They may have a potential role as simple screening or complementary tools, particularly in settings where access to advanced imaging is limited. Larger prospective multicenter studies are needed to validate the cut-off values and determine the clinical usefulness of these indices in different pediatric populations.

## Figures and Tables

**Table 1:** Comparison of selected anthropometric, biochemical, biochemical-anthropometric indices of liver steatosis between the examined groups.

Parameter	MASLD Group (n = 72) Median (Q1–Q3)	Non-Hepatopathic Controls (n = 31) Median (Q1–Q3)	*p*
Anthropometric			
Age (yrs)	12 (10–14)	12 (9–13)	NS
BMI (kg/m^2^)	29 (26.3–32.6)	26.1 (24.8–29.6)	0.008
Waist circumference (cm)	101 (91–106)	91 (82–97)	<0.01
Hip circumference (cm)	104 (96–113)	96 (92–102)	<0.01
WHR	0.95 (0.91–0.99)	0.92 (0.86–0.98)	NS
Biochemical			
ALT (U/L)	37 (23.5–62.5)	19 (13–26)	<0.001
AST (U/L)	29.5 (23–38.5)	22 (20–28)	<0.001
GGT (U/L)	22 (17–30)	16 (13–21)	<0.001
Bilirubin (mg/dL)	0.45 (0.33–0.69)	0.46 (0.36–0.59)	NS
Cholesterol (mg/dL)	153.5 (129.5–180)	157 (143–170)	NS
HDL-cholesterol (mg/dL)	45 (41.5–51)	45 (39–53)	NS
LDL-cholesterol (mg/dL)	97 (73–113)	99 (83–112)	NS
TG (mg/dL)	99 (78–132.5)	83 (62–133)	NS
Glucose (mg/dL)	86 (82–89.5)	86 (84–89)	NS
Insulin (µIU/mL)	21.4 (14.9–27.5)	14.6 (9.7–19.7)	<0.001
UC (µmmol/L)	5.85 (5.05–6.7)	5 (4.4–6.1)	0.009
HOMA-IR	4.65 (3.16–5.9)	3.07 (1.9–4)	<0.001
Biochemical-anthropometric indices of hepatic steatosis			
HSI	40.45 (36.8–44.3)	33.7 (32.1–39)	<0.001
LAP	40.5 (27–60)	27.4 (18.7–41.5)	0.005
FLI	58 (33–76)	26 (17–50)	<0.001

Footnote: MASLD—metabolic dysfunction associated steatotic liver disease, NS—statistically not significant, BMI—body mass index, WHR—waist-to-hip ratio, ALT—alanine aminotransferase, AST—aspartate aminotransferase, GGT—gamma-glutamyltransferase, HDL—high-density lipoprotein cholesterol, LDL—low-density lipoprotein cholesterol, TG—triacylglyceride, UC—uric acid, HOMA-IR—homeostasis model assessment score, HSI—hepatic steatosis index, LAP—lipid accumulation product, FLI—fatty liver index, CAP—controlled attenuation parameter.

**Table 2:** Univariate and multivariate analyses identifying independent predictors of hepatic steatosis among overweight/obese children.

Variable	Odds Ratio (95% C.I.)	*p*
**Univariate**		
HSI	1.215 (1.100–1.343)	<0.001
LAP	1.029 (1.005–1.054)	0.01
FLI	47.068 (6.641–333.574)	<0.001
Insulin	1.101 (1.035–1.171)	0.002
HOMA-IR	1.517 (1.517–1.990)	0.003
UA	1.650 (1.125–2.419)	0.01
**Multivariate**		
HSI	1.152 (1.026–1.292)	0.016
LAP	1.007 (0.984–1.031)	0.538
HOMA-IR	1.217 (0.923–1.603)	0.164
UA	1.233 (0.786–1.936)	0.362

HSI—hepatic steatosis index, LAP—lipid accumulation product, FLI—fatty liver index, UA—uric acid, HOMA-IR—homeostasis model assessment score.

## Data Availability

The datasets generated and analyzed during the current study are available from the corresponding author on reasonable request due to patient confidentiality.

## Abbreviations

WHO	World Health Organization
MASLD	Metabolic dysfunction-associated steatotic liver disease
CAP	Controlled Attenuation Parameter
AST	Aspartate aminotransferase
ALT	Alanine aminotransferase
GGT	Gamma glutamyltransferase
FLI	Fatty Liver Index Lipid Accumulation Product
HSI	Hepatic Steatosis Index
LAP	Lipid Accumulation Product
HCV	Hepatitis C Virus
HBV	Hepatitis B Virus
BMI	Body mass index
WC	Waist circumference
HC	Hip circumference
WHR	Waist-to-hip ratio
UA	Uric acid
TG	Triglycerides
HDL-C	High-density lipoprotein cholesterol
LDL-C	Low-density lipoprotein cholesterol
HOMA-IR	Homeostasis model assessment of insulin resistance
NAFLD	Nonalcoholic fatty liver disease
MAFLD	Metabolic dysfunction-associated fatty liver disease
AASLD	American Association for the Study of Liver Diseases
UDFF	Ultrasound-derived fat fraction
LUTE	Liver ultrasound transient elastography
AUC	Area under curve
NHANES	National Health and Nutrition Examination Survey
95%CI	95% confidence interval
